# Costs of polish county hospitals–A behavioral panel function

**DOI:** 10.1371/journal.pone.0262646

**Published:** 2022-01-18

**Authors:** Agata Sielska

**Affiliations:** Department of Applied Economics, Collegium of Management and Finance, Warsaw School of Economics SGH, Warsaw, Poland; LUMSA: Libera Universita Maria Santissima Assunta, ITALY

## Abstract

In the paper the costs of Polish county hospitals in 2015–2018 are studied using behavioral cost function. The set of variables combines hospitals’ characteristics which may determine their level of costs, such as the form of ownership, bed turnover rate, number of patient-days and share of beds in emergency department with environment characteristics which may influence both outsourcing costs and patients’ health. In 2017 the system of basic hospital service provision (hospital network) was introduced in Poland. Dummy variables included in the model represent the category of hospital in the system. The results show that the costs may be described using fixed effect panel model. Positive impact of percentage of emergency department patients transferred to other departments and of wages is found. Higher ratio of residents and interns to doctors is found to decrease costs. Dummy variable for the period after the introduction of hospital network assumed a negative sign with costs, but the parameter remained insignificant.

## Introduction

Specifics of the hospitals services does not release the managers from pursuing efficiency and avoiding wasting resources. [1] emphasizes the role of costs in the decision-making which takes place within management of hospitals, presenting also results of the questionnaire related to the approaches to costs calculations and usage of costs-related information by managers. Such information is useful mainly in budgeting and planning, costs control and profitability analysis. What is worth mentioning, the paper points out to the insufficient degree of the usage of this information in negotiations with the payer. Due to the fact that hospitals’ income is usually fixed in a way, costs are the only determinant which may be used by decision-makers in order to improve hospitals’ financial results [2].

In Poland the problem of cost containment in healthcare sector seems especially important due to the combination of unfavourable factors, i.a.: system focused around hospitals, overcapacity, low outpatient care [3], relatively low spending on healthcare, changes in the legal environment, lack of personnel and increasing debts[4].

Apart from some minor changes (e.g. employment norms for hospital nurses introduced in 2019 [4]), latest big reform of healthcare system in Poland was the introduction of the system of basic hospital service provision (hospital network) in 2017 [5]. Hospitals in the network have guaranteed financing from National Health Fund (polish: Narodowy Fundusz Zdrowia, NFZ) on lump sum basis. They are classified into one of seven categories which are summarized by [3].

Costs analyzed in the current paper result from operating activity and are the sum of amortization and depreciation, materials, energy, external services (medical and non-medical), payroll, social security and other benefits, taxes and charges and other costs by type.

Apart from the cost models strongly based on microeconomic theory, which use outputs and prices, in the literature one can find also behavioral models, which include variables related to various characteristics of the hospital [6, 7]. There are also hybrid models [7–9], which contain variables from both of those groups: production-related and non-production characteristics. The model presented in the current paper may be considered as belonging to the category of behavioral models. The goal is twofold: firstly, to describe hospital costs using econometric approach which was beforehand successfully implemented in hospital studies in the western literature (i.a. [10–13]). Secondly, the effect of the hospital network on costs is taken into account. It is verified whether hospital costs went down after inclusion in the network.

This approach is adopted due to several reasons. Firstly, the dataset obtained by the author is limited and the most data is aggregated by accounting categories. Secondly, in the current paper the goal is to estimate a cost functions that help to describe and model the costs. The objective is not to discuss whether hospitals are cost-minimizers and act at the optimum point. Additionally, due to the abovementioned changes which influence both the hospitals and their environment it is doubtful that during the analyzed period hospitals may be assumed to perform at optimum level.

The paper is organized as follows. The next part presents dataset, variables and model. Third section provides estimation results, while comments and discussions belong to the fourth part. The paper is concluded with summary, which states the conclusions drawn from the analysis and limitations.

## Materials and methods

### Data

Dataset used in the current paper does not include many of categories suggested by Kurup in the ideal approach [7], because it has been created for different reasons. Despite that fact different cost categories are available which allows for calculation of costs related to operation and the output.

Data used in the current study were provided by the Polish Association of Employers of Poviat Hospitals (OZPSP–Ogólnopolski Związek Pracodawców Szpitali Powiatowych), which associates hospitals that belong to Polish counties (powiats/poviats, i.e. the second-level units of local government and administration in Poland). Hospitals belonging to the Association gathered in 2019 data on their financial status during 4 previous years using questionnaire focused mostly on the accountancy data.

Due to the fact that the questionnaire was voluntary, there are some cases of missing data and ambiguous answers. After deleting such cases regarding the variables used in the study and converting the dataset into a balanced panel 48 hospitals remained in the sample.

Unfortunately, there are no data concerning DRG present in the dataset, which leads to the inability of DRG-weighting some of variables used in a model.

As mentioned before, costs resulting from hospital operating activity which are being analyzed are defined by the sum of amortization and depreciation, materials, energy, external services (medical and non-medical), payroll, social security and other benefits, taxes and charges and other costs by type. For the sake of simplicity they are referred to further simply by costs and denoted by *C*.

Data on the county (powiat/poviat) characteristics (outpatient medical advice per 10 000 population, share of population over 70, average gross monthly remuneration, number of beds, population density per 1 square kilometre, doctors per 10 000 population and nurses per 10 000 population) were taken from the Local Data Bank of the Statistics Poland [14].

Average structure of costs (*C*), i.e. means and medians of percentage share of a given category in costs is presented in Table 1. Considering the composition of costs, it may be noticed that in all years payroll has the highest median share, followed by medical services and materials. The same is true for means.

**Table 1:** Means and medians of percentage share of a given category in costs (*C*).

	2015		2016		2017		2018	
	Me	$\bar{x}$	Me	$\bar{x}$	Me	$\bar{x}$	Me	$\bar{x}$
Amortization and depreciation	5.276	5.535	5.363	5.544	4.661	4.986	4.573	4.882
Materials	14.976	15.605	14.559	15.369	14.377	15.104	13.647	14.358
Energy	2.207	2.287	2.0617	2.1336	1.9951	1.9986	1.8807	1.8505
External services (medical)	21.601	23.275	21.877	23.572	21.732	23.6	23.15	24.58
External services (non-medical)	6.071	6.07	6.08	6.099	5.961	6.313	5.9	6.201
Payroll	37.75	38	37.62	38.16	39.32	38.93	39.55	39.35
Social security and other benefits	7.886	7.6825	7.8166	7.6301	7.8569	7.6974	7.5676	7.5795
Taxes and charges	0.378	0.637	0.3628	0.5756	0.4541	0.5992	0.38549	0.52766
Other costs by	0.818	0.908	0.8412	0.9151	0.65751	0.7708	0.5812	0.6716

Values in percent’s. $\bar{x}$ stands for mean, Me–median. Values in columns do not sum up to 1 due to the fact that they represent means and medians of shares. Calculated based on the data from Polish Association of Employers of Poviat Hospitals.

The choice of explanatory variables was based on the literature study. The following section describes factors taken into account.

### Variables

#### Hospital performance

To the best of author’s knowledge there is no unambiguous way of measuring hospital output. There are different medical conditions which also are the function of patients’ and their environment characteristics, and the quality of the care belongs to the subjective sphere, even though in the literature various proxies for quality are used: nurses per beds ratio [15], readmissions [16, 17] and determinants of quality such as: HHI and buyer income [18].

There are different output variables in the models, usually determined by the research question: [16] use total number of inpatient spells and outpatient attendances, [17] use total number of admissions, day cases and outpatient visits (DRG values), similarly [19] use DRG-weighted inpatients and outpatients. Inpatient days are employed in [18], inpatient discharges, outpatients visits and average length of stay in [12], and patient-days in [20]. In [21] it is argued that patient days are better measure of output since the costs are connected to the number of days patients spend in a hospital. In the current study patient-days is used due to the fact that discharges are aggregated in the dataset with deaths which could lead to the inclusion of undesirable output in the model.

Apart from the patient-days Bed Turnover Rate Average (BTRA), calculated by formula (1) is taken into account.

$$BTRA=\frac{DISDEAD}{TOTBEDS}∙100$$ (1)

where:

DISDEAD–number of patients (discharged and dead),

TOTBEDS–total number of beds in a hospital.

Emergency activity is taken into consideration in [15] and in [22] emergency department is seen to have an impact on costs. Emergency beds and emergency admissions were included in [11] as well. In the questionnaire completed by county hospitals from which data for the current study is taken, there was no direct question whether an Emergency Department (further referred to as ED) or intensive care unit (further referred to as ICU) function in the hospital. Therefore it is assumed that the hospital has these units if it reports a non-zero number of emergency beds or ICU beds, respectively.

In a similar way as in [23], the source of admission is included in the model in form of P.ED variable representing the ratio of patients transferred from ED to other departments to the number of patients admitted in ED (in case of hospitals with no ED this is assumed to be equal to 0).

Teaching status [9–13, 17, 20, 23, 24] is addressed only indirectly, because there are no university hospitals or clinics in the panel. On the other hand, due to the fact that majority of hospitals employ doctor interns and/or residents, a variable representing the ratio of them to doctors was created in order to verify the impact of that personnel on costs.

Based on the literature, in the current study binary variable for non-public (commercialized) hospitals is used. Adam et al. [6] used dummies for ownership in their cross-country study, dummy for government owned hospitals is used by [21], dummies for different types of hospitals are used in [9, 20]. Similarly [18] took into account different types hospitals–estimating the functions separately. The role of ownership is discussed in detail in [25].

Number of beds and contract with National Health Fund which may be used to represent the size of hospital were to be included in the model, but after checking for multicollinearity both were omitted due to the high correlation with patient-days (i.e. the output variable).

#### Category in the network

After clearing the data all hospitals in the balanced panel were part of the hospital network. The number of security system profiles (polish: profile systemu zabezpieczenia) which are covered by the network is not included in the dataset, hence the level of security system is used to represent approximately complexity of the hospital and case mix. This is represented in two ways: firstly, binary variables corresponding to each level are introduced. They are assumed to reflect the general complexity and case mix in a hospital (similar proxy was used by [18] in the form of the number of accreditations). Secondly, additional two binary variables for each level in 2017 and 2018 are present to reflect the change in the first and the second year of the hospital network. Year 2017 was the first year in which the hospital network was active, therefore it was decided to differentiate it from 2018. The registers of hospitals included in the network were to be published for each province (voivodeship, i.e. the highest-level unit of local government and administration in Poland) before the end of June 2017 and they are in force from October 1^st^ 2017 [5].

#### Wages and employment

Wages of both doctors and nurses [17] on an annual basis are approximated by the ratio of personnel costs and contracts to the level of employment of a respective group. Due to the fact that many doctors and nurses work in more than one health facility at the same time, in the current paper full-time equivalent is used to measure the employment of doctors and nurses instead of the number of people in the respective personnel group. This is only one of the ways in which wages may be introduced to the model. [18] use average employee wage determined by dividing the total payroll by the number of employees (with some exclusions), in [11] wage index is used.

In the current study only two groups of personnel are taken into account while calculating wages, while in the literature other professionals are taken into account as well: [23] included variable representing nonphysician wage per fulltime equivalent employee, [17] also use variable called OTHERS WAGE, but it is not included in the current paper, due to the fact that not all hospitals provided data related to the employment of other groups of personnel. The author decided for including more hospitals and omitting this variable. Wages of other groups are represented in a model by a RENUM variable, which represents the average monthly renumeration in a county in which a hospital is located. It may also approximate the costs of outsourcing, which is popular in Polish hospitals as a method of saving [26].

#### Environment

In the literature variables related to the environment include: Herfindal-(Hirschman) Index [9, 12, 18, 23], income [13] or GDP per capita (which was included by [6] in cross-country study), population density [13], doctors per population [13], unemployment rate [13].

Even though it may be expected that county hospitals are similar, because they were founded by the territorial units of the same level, there are differences in their structure and in environments in which they are operating. Hospitals located in small towns and satisfying the demand for health services of the local villages are faced with different expectations and challenges than those located in cities which are counties in their own right. This is approximated and addressed by the population density and average remuneration (RENUM). The reason for including population density was explained by [9] who, following [23] stated that urban hospitals are more costly.

In [9] it is stated that greater competition leads to higher costs, thus it should be addressed in the model. In the literature market situation is proxied in different ways, e.g. [16] use the number of hospitals in proximity, although it seems that the approach with Herfindal index as a proxy for market is very popular.

In the current paper market position is proxied by Herfindahl-Hirschman Index (HHI) in provinces calculated based on beds, following [27, 28]:

$$HHI=\sum_{i=1}^{n}sbeds_{i}^{2}$$ (2)

where:

sbeds_i_−share of beds of county i,

n–number of counties in a province.

Environment variables in the current study are used to describe not only market conditions, but the health status of population. As mentioned in [23] treating low income patients may result in higher costs since those people may suffer from additional health issues. Thorpe [23] relates this phenomenon to the share of patients admitted via ED and although similar variable is also used in the current paper, it was decided to use more characteristics of environment and potential patients. Number of doctors and nurses per 10 000 population and medical advices per capita as well as the share of population over 70 are included. It is expected that better out-of-hospital medical care and lower share of most vulnerable population should translate into lower costs.

### Model specification

In the paper panel models are used following [10, 16, 24].

Hospital costs are usually modelled using Cobb-Douglas [10, 15, 18, 19] or translog functions [10, 19, 20, 29]. Both are linear in parameters which facilitates the estimation, because non-linear methods or optimization algorithms are not needed. The Cobb-Douglas form is preferred especially in smaller datasets due to the fact that it requires less parameters. In [15] it is also pointed to the possible collinearity problems which may result from squared variables in translog model. On the other hand such specification also involves assumption on the constant and equal 1 elasticity of substitution which may be considered too rigorous in some cases. It is pointed out, that in the literature there is no agreement about the form of hospital cost function [7, 24]. Flexible functional forms are discussed by [30]. Thorpe [23] uses semilog specification, [19] used quadratic function as well, Vitaliano [31] used different specifications which included squares of explanatory variables and logarithm of costs, and Li and Rosenmann [21] used generalized Leontieff.

In the paper semilog function is used following [23]. All the variables are in their logs apart for dummies and non-binary variables representing shares which are allowed to be 0. The general form of a model is presented in Eq (3). Natural logarithms are used.


$$\ln\left(C\right)=\ln\left(\alpha_{0}\right)+\alpha_{1}ln(Y)+\sum_{i=2}^{n_{1}+1}\alpha_{i}lnX_{i}+\sum_{i=1}^{n_{2}}\beta_{i}Z_{i}$$ (3)

Where: C–costs, Y–output, X–vector of continuous variables capturing hospital and environment characteristics, Z—the vector of dummy variables and shares capturing hospital and environment characteristics.

The cost function is specified as (for the sake of simplicity time and observation indices are omitted in the formula):

$$\ln\left(C\right)=\ln\left(\alpha_{0}\right)+\alpha_{1}\ln\left(PATD\right)+\alpha_{2}\ln\left(BTRA\right)+\alpha_{3}COMMER+\alpha_{4}ED+\alpha_{5}ICU+\alpha_{6}P.ED+\alpha_{7}ln(MEDAD)+\alpha_{8}PERC70+\alpha_{9}\ln\left(WDOC\right)+\alpha_{10}\ln(WNURS)+\alpha_{11}P.TEACH+\alpha_{12}\ln(RENUM)+\alpha_{13}\ln\left(HHI\right)+\alpha_{14}\ln\left(DENS\right)+\alpha_{15}\ln\left(DOC\right)+\alpha_{16}\ln\left(NURS\right)+\alpha_{17}AFTER2016+\alpha_{18}CAT.O+\alpha_{19}CAT2+\alpha_{20}CAT3+\alpha_{21}CAT.O.17+\alpha_{22}CAT2.17+\alpha_{23}CAT3.17+\alpha_{24}CAT.O.18+\alpha_{25}CAT2.18+\alpha_{26}CAT3.18$$ (4)

Where:

C–costs (sum of amortization and depreciation, materials, energy, medical and non-medical external services, payroll, social security and other benefits, taxes and charges and other costs by type),

PATD–patient-days total,

BTRA–Bed turnover rate calculated as in formula (1)

COMMER–dummy for non-public hospitals,

ED–share of beds in an emergency department in the total number of beds,

ICU–share of beds in an ICU in the total number of beds,

P.ED–share of patients transferred from ED to other departments/wards in the total number of patients admitted in ED,

MEDAD–medical advice per 10 000 population in county,

PERC70 –share of population over 70 in county,

WDOC–doctors’ wage (approximated by the ratio of personnel costs and contracts to the level of employment calculated for doctors),

WNURS–nurses’ wage (approximated by the ratio of personnel costs and contracts to the level of employment calculated for nurses),

P.TEACH–ratio of doctor interns and residents to the number of doctors,

RENUM–average monthly remuneration in county,

HHI–Herfindal-Hirshfeld index for province (formula (2)),

DENS–population density (per 1 sq kilometer) in county,

DOC–doctors per 10 000 population in county,

NURS–nurses per 10 000 population in county,

AFTER2016 –dummy for years 2017 and 2018,

CAT.O–dummy for hospitals with category “other” in the hospital network,

CAT2 –dummy for hospitals with category “2” in the hospital network,

CAT3 –dummy for hospitals with category “3” in the hospital network,

CAT.O.17 –dummy for hospitals with category “other” in the hospital network and 2017,

CAT2.17 –dummy for hospitals with category “2” in the hospital network and 2017,

CAT3.17 –dummy for hospitals with category “3” in the hospital network and 2017,

CAT.O.18 –dummy for hospitals with category “other” in the hospital network and 2018

CAT2.18 –dummy for hospitals with category “2” in the hospital network and 2018,

CAT3.18 –dummy for hospitals with category “3” in the hospital network and 2018.

The comparator for categories in the hospital network is category 1 (i.e. the lowest of the three basic categories).

Some descriptive statistics of the final panel are presented in Table 2.

**Table 2:** Descriptive statistics of variables.

	2015		2016		2017		2018	
	Me	$\bar{x}$ (Sd)	Me	$\bar{x}$ (Sd)	Me	$\bar{x}$ (Sd)	Me	mean (Sd)
Total number of beds	254.5	276.9 (132.3276)	257	276.4 (132.41)	245.5	276.4 (132.3549)	237	274.4 (131.7323)
Beds in emergency department	3.5	3.438 (3.5305)	3.5	3.562 (3.7068)	3.5	3.646 (3.7614)	4	3.896 (4.0225)
Beds in ICU	5	4.833 (2.4086)	5	4.938 (2.555)	5	5 (2.6175)	5	5.125 (2.8029)
Patient-days total	55961	67865 (37264.13)	57155	67592 (36758.34)	58446	68454 (37139.38)	54779	66686 (36716.44)
Number of patients (discharged and dead)	10566	13325 (9137.856)	10347	13487 (9405.062)	9748	13487 (9568.631)	9962	13367 (9421.556)
C	37070808	48576389 (30414109)	38398260	51637720 (32153196)	42107682	55277639 (34469194)	46412158	61985050 (38639514)
Doctors (costs and contracts)	7379608	9130142 (6427512)	7793289	9525822 (6819825)	7984260	10015385 (7151761)	8865750	11473762 (8214750)
Nurses (costs and contracts)	7983524	10133858 (6337849)	9036313	11241171 (7010416)	9825221	12462321 (7730788)	11117412	14364554 (8991390)
Doctors (full-time equivalent)	58.31	141.91 (338.957)	58.65	146.53 (356.9874)	58.22	146.29 (358.5195)	59.51	144.79 (350.9298)
Nurses (full-time equivalent)	199.49	459.76 (991.9734)	200.35	457.6 (997.3852)	201.3	458.3 (972.4061)	197.9	462.4 (984.7814)
DENS	96.5	355.6 (721.3955)	96.5	355.5 (722.1943)	96.5	355.4 (722.8304)	96.5	355.3 (723.8318)
RENUM	3363	3500 (486.5718)	3492	3629 (487.6155)	3682	3845 (509.7439)	3953	4108 (547.2154)
DOC	30.25	39.85 (29.5567)	29.35	41.16 (32.1087)	30.25	41.17 (31.7543)	31.5	42.81 (32.7099)
NURS	50.7	57.9 (28.535)	52.15	58.61 (30.196)	52.65	61.36 (30.2854)	54.15	63.09 (30.7419)
HHI	0.1684	0.1742 (0.0463)	0.1627	0.1759 (0.0497)	0.166	0.1764 (0.0492)	0.171	0.1776 (0.0502)
PERC70	0.0958	0.0979 (0.0159)	0.0983	0.1008 (0.0155)	0.1009	0.1042 (0.0155)	0.1034	0.108 (0.0157)
MEDAD	64403	65833 (17425.03)	63616	66865 (17565.31)	64082	66909 (18015.49)	64956	67722 (18400.98)
P.ED	12.05	12.43 (14.0721)	6.768	12.124 (14.1494)	11.35	12.49 (14.0154)	13.35	13.51 (14.4722)
BTRA	4250	4894 (2091.718)	4278	4959 (2198.531)	4342	4935 (2226.833)	4113	4948 (2229.129)
Doctor interns (full-time equivalent)	1	5.977 (14.1767)	2	6.33 (14.7238)	1.45	6.843 (16.1635)	1.205	8.082 (19.0204)
Residents (full-time equivalent)	6.51	18.9556 (57.5576)	6.18	17.71 (43.0907)	7.42	19.36 (51.1877)	7.51	21.97 (59.5424)
P.TEACH	0.1069	0.1567 (0.1688)	0.1172	0. 1927 (0.2294)	0.15	0. 19351 (0.2047)	0.1481	0.2506 (0.3929)

SD stands for standard deviation, $\bar{x}$–mean, Me–median. Calculated based on the data from Polish Association of Employers of Poviat Hospitals and from Local Data Bank of the Statistics Poland [14].

In the description below medians (Me) are used. Depending on the year, the average hospital has 237–257 beds out of which 3.5–4 belonged to ED and 5 to ICU. It is characterized by 54779–58446 patient-days, with BTRA 4113–4342, employs (in terms of full-time equivalents) 58.22–59.51 doctors, 197.9–201.3 nurses, 1.2–2 doctor interns and 6.18–7.51 residents. Median operations costs are equal from 37070808 to 46412158.

Due to the fact that the majority of hospitals employed at least one intern or resident, and taking into account that as shown by the median values of P.TEACH–the median ratio of interns and residents reached from 10.69% to 15% percent of doctors (all in terms of full-time equivalent) it seems that defining teaching hospital as one that employs interns or residents is inadequate. Therefore P.TEACH variable was used. Most hospitals belonged to category 1–3 (i.e. basic ones, thought for hospitals operating on the local or regional level) in the network. Only one was classified as “other”. 9 out of 48 hospitals are non-public, 27 have ED (however one introduced it in 2018), almost all (43) have an ICU, 37–39 employ residents and 29–35 employ doctor interns.

### Software

Study was conducted using R [32] and packages PLM [33, 34], regclass [35], lmtest [36] and sandwich [37–39].

## Results

VIFs (Variance inflation factors) calculated for the random effects (RE) and pooled model (of an identical structure as RE, given by formula (4)) in which dummies for category 1 instead of “other” are present, lead to the conclusion that multicollinearity was present (at the threshold of 10 [40, 41]). After changing the specification and including category 1 as a comparator instead of “other” VIFs showed no evidence of multicollinearity as presented in Table 3.

**Table 3:** Variance inflation factors for RE and pooled model.

	RE	pooled
Ln(PATD)	1.6967	2.9795
Ln(BTRA)	1.3724	2.0386
ICU	1.8062	1.8077
ED	1.6919	3.0562
P.ED	1.8995	3.6053
COMMER	1.244	1.7113
Ln(MEDAD)	1.4495	4.2216
PERC70	4.8567	3.1082
Ln(WDOC)	1.9927	2.8721
Ln(WNURS)	3.739	2.9564
P.TEACH	1.188	1.9022
Ln(RENUM)	8.9867	4.2639
Ln(HHI)	1.2254	1.3741
Ln(DENS)	2.1951	3.8686
Ln(DOC)	2.0941	8.2378
Ln(NURS)	2.3851	5.8824
AFTER2016	5.7757	2.1921
CAT.O.17	1.196	1.5284
CAT.O.18	1.1995	1.5255
CAT2.17	1.444	1.7022
CAT2.18	1.5544	1.717
CAT3.17	1.1719	1.5246
CAT3.18	1.1825	1.5244
CAT.O	1.7131	3.4179
CAT.2	1.7674	3.8244
CAT.3	1.4137	2.7246

Calculated based on the data from Polish Association of Employers of Poviat Hospitals and from Local Data Bank of the Statistics Poland [14].

Table 4 presents estimation results for RE and fixed effects (FE) models defined by Eq (4). α = 0.05 is assumed for the interpretation in the whole paper.

**Table 4:** Estimation results.

	RE		FE	
	Coefficient and standard error	p-value	Coefficient and standard error	p-value
(Intercept) Intercept	2.3480738 (1.7040755)	0.170095		
ln(PATD)	0.3751406 (0.0902643)	5.19E-05	0.0785997 (0.0733874)	0.28627
ln(BTRA)	0.0829902 (0.0580614)	0.154794	0.1535709 (0.0795342)	0.05582
ICU	0.0189309 (0.0247736)	0.445867	-0.0169407 (0.028693)	0.55601
ED	0.0044689 (0.0086903)	0.607775	-0.0062291 (0.0144357)	0.66686
P.ED	0.0036042 (0.0012033)	0.003165	0.0025834 (0.0012927)	0.0479
COMMER	-0.0421973 (0.0965432)	0.662623		
ln(MEDAD)	-0.0586366 (0.0650921)	0.368994	0.0015644 (0.0869725)	0.98568
PERC70	2.0341925 (1.7364945)	0.243112	3.8211969 (1.9644166)	0.05405
ln(WDOC)	0.0339687 (0.0278649)	0.224565	0.0659358 (0.0326994)	0.04595
ln(WNURS)	0.0332046 (0.0553487)	0.549384	0.127914 (0.0640169)	0.04793
P.TEACH	-0.0303882 (0.0159686)	0.058783	-0.0474345 (0.0220121)	0.03313
ln(RENUM)	1.1853186 (0.1762448)	<0.00001	0.9111567 (0.1757553)	<0.00001
ln(HHI)	0.0593597 (0.1141323)	0.603694	-0.0031337 (0.1695846)	0.98529
ln(DOC)	0.0570199 (0.0338666)	0.094138	0.0832674 (0.0411711)	0.04531
ln(NURS)	0.0502609 (0.0497183)	0.313538	0.060927 (0.0538326)	0.25994
ln(DENS)	-0.0059788 (0.0485919)	0.902224	0.4794679 (0.5575856)	0.39153
AFTER2016	-0.0046033 (0.01348)	0.733168	-0.0085272 (0.0143226)	0.5527
CAT.O.17	0.0860178 (0.0104844)	<0.00001	0.0745732 (0.0505324)	0.14259
CAT.O.18	0.1250368 (0.0114545)	<0.00001	0.1194377 (0.0518214)	0.02287
CAT2.17	-0.0260759 (0.0092753)	0.005532	-0.0187397 (0.017604)	0.2892
CAT2.18	-0.0120326 (0.0200819)	0.549875	-0.0066055 (0.018812)	0.7261
CAT3.17	-0.0232853 (0.0082783)	0.005508	-0.019282 (0.0491269)	0.69538
CAT3.18	0.0094024 (0.0108618)	0.387946	-0.0009439 (0.0496071)	0.98485
CAT.O	-0.5910276 (0.2233976)	0.008942		
CAT2	0.4375231 (0.118876)	0.000315		
CAT3	0.6769734 (0.1734127)	0.000138		
Breusch-Pagan test	BP = 33.864, df = 26, p-value = 0.1385		BP = 33.864, df = 26, p-value = 0.1385	
Pesaran CD test	z = 1.6212, p-value = 0.105		z = 0.02268, p-value = 0.9819	
Wooldridge’s test	-		F = 0.60491, df1 = 1, df2 = 142, p-value = 0.438	
Breusch-Godfrey/Wooldridge test	chisq = 9.5228, df = 4, p-value = 0.04928		-	

Time-invariant dummies are excluded from FE regression. Standard errors are presented in brackets. VCX estimator robust vs. heteroskedasticity and serial dependence is used for RE model [34]. For FE model Wooldridge’s test for short series is used. α = 0.05 is assumed for the interpretation. Calculated based on the data from Polish Association of Employers of Poviat Hospitals and from Local Data Bank of the Statistics Poland [14].

At α = 0.05 FE model does not exhibit problem with autocorrelation, heteroscedasticity or cross- dependence and adjusted R-squared equal to 0.83723 suggests a good fit. When hospitals’ characteristics are concerned, there is a positive impact of percentage of patients admitted in ED who are transferred to other departments, other category in 2018, wages of doctors and nurses on costs. In case of variables describing the environment, the positive effect is significant only for average remuneration and ratio of doctors per 10 000 population. The impact of P.TEACH is significantly negative.

At α = 0.05 RE does not exhibit problem with heteroscedasticity or cross-sectional dependence. Due to the presence of autocorrelation, robust estimators are presented in Table 4. Similarly to the FE model, adjusted R-squared (0.83267) suggests a good fit. Patient-days total, share of patients transferred from ED, average remuneration, category 2 and 3 (in general) as well as other category in 2017–2018 significantly increase costs. On the other hand only dummies for second and third category in 2017 and “other” category show a statistically significant negative impact on costs.

Hausman test was used for comparison of FE and RE model. Test statistic equal to 53.646 (22 degrees of freedom and p-value 0.00018) suggest that FE model is an appropriate one.

## Discussion

Current paper expands the literature on Polish hospitals. To the best of the author’s knowledge, there were so far no other attempts to estimate costs for county hospitals in Poland. In [42] linear operation cost function was estimated, but based only on one hospital (time period of 21 months was used in the estimation). Publications were focused mostly on general rules and specifics of cost accounting in hospitals [43], indirect costs [44] or determinants of costs accountancy, e.g. [45] focused on the relation between the computer system in hospital and quality of costs accountancy, and the level at which information coming from costs is used in management.

As compared with other models of hospital costs functions, the current paper does not use length of stay [9, 10, 12, 18, 24] and patients are not divided per payment category (out-of pocket vs reimbursed later by third party). Similarly, case mix [9, 13, 23, 24] is not directly included. This may be considered a downside as this variable turned out to have a significant impact on costs [9]. The reason is that the current dataset does not contain such an information.

Analyzing conclusions drawn from RE and FE models presented in the previous section in may be noticed, that some of the previous findings reported in the literature are confirmed in the case of Polish county hospitals. Firstly, the relation between the output and costs is obviously positive (significant only for RE model). [12] reports positive sign of respective parameters in his paper. It should be noted, that the relation may in fact be nonlinear, as suggested by Carey [8, 24] for discharges, but adding the square of the patient-days to the model in the current study resulted in multicollinearity.

Secondly, the relation between wages and costs is clear and obvious. Higher wages are one of the most important determinants of costs, especially of operating costs, which include hospitals’ personnel expenditures. This is confirmed by positive signs of parameters reported in [10, 11, 12, 18, 23]. In both models used in the current paper doctors’ and nurses’ wages assume positive signs, although parameters are significantly different from 0 only in the case of FE model (which, according to Hausman test is the better one).

Literature suggests that emergency services increase costs [10, 11, 23], although in some cases this impact is not significant [10]. In case of Polish county hospitals it was suggested in the preliminary analysis in [22] but no controls were included in the cited report. What is even more interesting is the fact that not the share of beds in ED is important, but the share of people who are admitted in ED and then transferred to other wards/departments (variables representing share of beds are not significantly different from 0 for FE and RE, what is more the respective parameter is positive only in case of RE). This was previously suggested in [23].

COMMER assumes a negative sign, however it is not significantly different from 0. From the one point of view this result may be surprising taking into account the fact that ownership was found to have an impact on costs [6], on the other side this may be included in the effect of an Emergency Department. As presented in Table 5 for pooled observations, the fact whether a hospital runs an ED or not is related to the ownership. Pearson’s Chi-squared test with Yates’ continuity correction statistics equals 7.1273 with a p-value of 0.0076, which at α = 0.05 leads to the conclusion of significant association between those two variables.

**Table 5:** Distribution of the number of hospitals by ownership and emergency department (pooled observations).

	No Emergency Department	With Emergency Department
Public (COMMER = 0)	63	93
Non-public (COMMER = 1)	24	12

Table presents pooled observations from panel in 2015–2018. Hospital is defined as having an emergency department if ED>0. Calculated based on the data from Polish Association of Employers of Poviat Hospitals.

In [18] a positive impact of the inclusion of a non-profit or government hospital in a health care system is reported. Although all hospitals in the current study are included in the network, the level on which they operate has a clear impact in RE model. The costs are lower in case of “other” category hospitals (for RE model). Dummies for this category and years 2017 and 2018 have positive impact in both models and, except for other category in 2017 and FE, are significant. On the other hand the estimate for dummy variable AFTER2016 does not significantly differ from 0. Based only on this fact one cannot say, that the introduction of hospital network lead to the decrease of costs.

Interns and residents have significantly negative impact on costs in FE model most likely due to the fact that their training in hospital is financed from the outside. They do not have the same permissions as full doctors, but may complete some of the tasks, reliving other personnel. Such benefits are seemingly greater than potential increase of costs due to rising demand for materials etc. used in training. This results are surprising in the light of the direction of the impact reported in the western literature [9–11], but it should be noted that none of the hospitals in the sample is a clinic run by a medical university and therefore, due to the different definition of how the teaching in a hospital is understood, they cannot be compared in a straightforward way.

In [6] GDP per capita and in [13] income assume positive signs with costs. In the current study their roles are taken up by the remuneration in the given county which may be used as a proxy for not only income (and therefore in a consequence also the level of health expense) but also for the prices of external services bought by hospitals on local markets, such as cleaning, catering, etc. Positive sign on this variable which appeared both in FE and RE model was to be expected. This influence is also significant.

According to [9] competition between hospitals should increases costs. In case of the competition for the payer funds, the hospitals which are included in the network have a privileged position as after the contract is signed, they do not need to enter a wager. Of course, there is also a possibility of over-performance, the costs of which are not covered by the payer (see [46]). In the current study in case of FE model the effect of HHI is negative but insignificant, while in the RE model it is positive, yet still insignificant. Comparing this with other findings one may notice that Herfindal index assumes negative sign with costs in [9].

The effect of the degree of urbanization of the regions in which hospitals are located is mixed in the literature. Dummy for urban market area assume a positive sign with costs for two samples in [18] where it is included mainly as a proxy for case mix. In [13] population density assumes a positive sign with costs. Effect of population density found in the current paper is insignificant (both in FE and RE).

Variables reflecting the health status of population may provide additional information. Estimates of parameters for DOC, NURS and MEDAD are in majority insignificant. The only one that is statistically different from zero is the estimate of DOC in FE model. Based on that it may be stated that higher ratio of doctors per 10 000 population increase hospital costs. That seems to contradict expectations, as patients who are better cared for were expected to be in better condition and in less need of hospital treatment. On the other hand such patients may be treated for longer time due to the fact that their (potentially severe) conditions diagnosed earlier. Another possible explanation is that large number of doctors and nurses goes in line with larger number of out-of-hospital medical services, which are often seen as a better employer by the personnel. Similarly as with HHI, the growing competition may result in difficulties for some hospitals and this is the explanation favoured by the author. It need to be taken into account, that this impact is in most cases insignificant. Positive sign of the parameter next to PERC70 –a variable associated with the share of population over 70 was to be expected, although the impact of this variable on costs is insignificant as well.

## Conclusion

The paper used panel methods to estimate the behavioral cost function for Polish county hospitals for the period: 2015–2018 based on the data of 48 hospitals. The results show that the presented approach is suitable for the problem and the costs may be described using fixed effect (FE) panel model. Positive impact of percentage of ED patients transferred to other departments and of wages is reported. Variables related to the local environment, i.a. ratio of doctors per 10 000 local population were found to increase costs as well. Finding regarding teaching activity is at variance with result previously reported by other authors, but one should keep in mind different definition of teaching hospital which was used in the current study based on the sample. Although a dummy variable for the period after the introduction of hospital network assumed a negative sign with costs, this relation is not statistically significant. The differences in costs with respect to category in the network were studied.

Despite abovementioned findings, it should be stressed that the study has several limitations. The current paper does not use some of the variables which turned out to be significant in other studies, due to the fact that information on length of stay, per payment category (out-of pocket vs reimbursed later by third party), ratio of out- to inpatient services and case mix. Boutsioli [11] emphasized the unexpected demand. It is also not possible to forecast admissions, so this determinant (also indirect one, because costs may be affected not only by the forecasts themselves, but by the ratio of forecasted to actual admissions [23]) is not included in the model.

## References

[1] Kludacz M. Rachunek kosztów i jego wykorzystanie w zarządzaniu szpitalem. Prace Naukowe Uniwersytetu Ekonomicznego We Wrocławiu. Research Papers of Wrocław University of Economics. 2015; 389: 160–171 doi: 10.15611/pn.2015.389.15

[2] Szewieczek A. Rachunek kosztów szpitala–analiza i ewolucja regulacji prawnych. Studia Ekonomiczne. Zeszyty Naukowe Uniwersytetu Ekonomicznego w Katowicach. 2019; 386: 108–121.

[3] Dubas-Jakóbczyk K, Kowalska-Bobko I, Sowada C. The 2017 reform of the hospital sector in Poland–The challenge of consistent design. Health Policy. 2019; 123(6): 538–543. Available from: doi: 10.1016/j.healthpol.2019.03.013 30940457

[4] Sowada C, Kowalska-Bobko I, Sagan A. What next after the ‘commercialization’ of public hospitals? Searching for effective solutions to achieve financial stability of the hospital sector in Poland. Health Policy. 2020; 124: 1050–1055. doi: 10.1016/j.healthpol.2020.05.024 32782112

[5] Ustawa z dnia 23 marca 2017 r. o zmianie ustawy o świadczeniach opieki zdrowotnej finansowanych ze środków publicznych. Dz.U. 2017 poz. 844 [Amendments to the Law on Health Care Services Financed from Public Sources] (Accessed 08 February 2021) http://isap.sejm.gov.pl/isap.nsf/download.xsp/WDU20170000844/T/D20170844L.pdf.

[6] Adam T, Evans DB, Murray CJ. Econometric estimation of country-specific hospital costs. [Internet] Cost Eff Resour Alloc. 2003 [Cited: 21 October 2020] Feb 26;1(3). doi: 10.1186/1478-7547-1-3 Available from: http://www.resource-allocation.com/content/1/1/3. 12773218 PMC156022

[7] Kurup HKK. On the estimation of hospital cost: the approach. MPRA Paper No. 22767. [Internet] 2010 [Cited: 22 Jan 2021]. Available from: https://mpra.ub.uni-muenchen.de/22767/.

[8] Carey K. Hospital Cost Containment and Length of Stay: An Econometric Analysis. South Econ J. 2000; 67(2): 363–380. doi: 10.2307/1061475

[9] Carey K, Stefos T. Measuring inpatient and outpatient costs: A cost function approach. Health Care Financ Rev. 1992; 14(2): 115–124. 10127447 PMC4193307

[10] Almeida AS, Cima JF. Demand uncertainty and hospital costs: an application to Portuguese public hospitals. Eur J Health Econ. 2015; 16(1):35–45. doi: 10.1007/s10198-013-0547-3 24310509

[11] Boutsioli Z. Hospital Costs and Unexpected Demand: The Case of Greece. The Open Economics Journal. 2011; 4:49–58. doi: 10.2174/1874919401104010049

[12] Schneider JE. Changes in the Effects of Mandatory Rate Regulation On Growth in Hospital Operating Costs, 1980–1996. Rev Ind Organ. 2003; 22(4): 297–312.

[13] Schneider JE, Ohsfeldt RL, Morrisey MA, Pengxiang L, Miller TR, Zelner BA. Effects of Specialty Hospitals on the Financial Performance of General Hospitals, 1997–2004. Inquiry. 2007; 44(3): 321–334. doi: 10.5034/inquiryjrnl_44.3.321 18038867

[14] Statistics Poland Local Data Bank [Internet]. 2021 Główny Urząd Statystyczny (Statistics Poland) [cited 2021 Jan 08]. Available from: https://bdl.stat.gov.pl/BDL/dane/podgrup/temat.

[15] Farsi M, Filippini M. Effects of ownership, subsidization and teaching activities on hospital costs in Switzerland. [Internet] 2007 [Cited: 2021 Jan 14]. Available from: http://citeseerx.ist.psu.edu/viewdoc/summary?doi=10.1.1.429.4995. 10.1002/hec.1268 17619236

[16] Siciliani L, Stanciolez A, Jacobsx R. Do waiting times reduce hospital costs? The University of York. Discussion Papers in Economics. No. 2008/02 [Internet].

[17] Vitikainen K, Linna M, Street A. Substituting inpatient for outpatient care: what is the impact on hospital costs and efficiency? Eur J Health Econ. 2010; 11(4): 395–404. doi: 10.1007/s10198-009-0211-0 20037768

[18] Santerre RE, Benett DC. Hospital Market Structure and Cost Performance: A Case Study. Eastern Economic Journal. 1992; 18(2): 209–219.

[19] Kristensen T, Olsen KR, Kilsmark J, Pedersen KM. Economies of scale and optimal size of hospitals: Empirical results for Danish public hospitals. University of Southern Danemark. Health Economics Papers 2008:13.

[20] Cowing TG, Holtmann AG. Multiproduct Short-Run Hospital Cost Functions: Empirical Evidence and Policy Implications from Cross-Section Data. South Econ J. 1983; 49(3): 637–653.

[21] Li T, Rosenman R. Estimating hospital costs with a generalized Leontief function. Health Econ. 2001; 10: 523–538. doi: 10.1002/hec.605 11550293

[22] Nojszewska E, Sielska A, Gołąb-Bełtowicz D. Raport z badania sytuacji finansowej szpitali powiatowych–Szklarska Poręba’19. [Internet] 2019 [Cited: 2020 Nov 30] Available from: http://ozpsp.pl/slider/raport-na-temat-sytuacji-szpitali-powiatowych/.

[23] Thorpe KE. Why Are Urban Hospital Costs So High? The Relative Importance of Patient Source of Admission, Teaching, Competition, and Case Mix. HSR: Health Services Research. 1988; 22(6): 821–836. 3126164 PMC1065477

[24] Carey K. A Panel Data Design for Estimation of Hospital Cost Functions. Rev Econ Stat. 1997;79(3): 443–453. doi: 10.1162/003465300556850

[25] Shen Y-C, Eggleston K, Lau J, Schmid CH. Hospital Ownership and Financial Performance: What Explains the Different Findings in the Empirical Literature? Inquiry. 2007; 44(1): 41–68. doi: 10.5034/inquiryjrnl_44.1.41 17583261

[26] Borowska M, Augustynowicz A, Bobiński K, Waszkiewicz M, Czerw A. Selected factors determining outsourcing of basic operations in healthcare entities in Poland. Health Policy. 2020; 124(4): 486–490. doi: 10.1016/j.healthpol.2020.01.010 32063379

[27] Łyszczarz B. Market concentration and performance of general hospitals in Poland. In: Polszakiewicz B, Boehlke J, editors. Ekonomia i Prawo. Economics and Law. 2014; 13(4): 499–510. doi: 10.12775/EiP.2014.035

[28] Rój J. Competition Measurement of Hospitals in Poland: the Herfindahl-Hirschman Index Approach. EKONOMIKA. 2016; 95(1): 166–181. doi: 10.15388/Ekon.2016.1.9912

[29] Keeler TE, Ying JS. Hospital Costs and Excess Bed Capacity: A Statistical Analysis. Rev Econ Stat. 1996; 78(3): 470–481.

[30] Vita MG. Exploring hospital production relationships with flexible functional forms. J Health Econ. 1990; 9(1): 1–21 doi: 10.1016/0167-6296(90)90038-5 10105280

[31] Vitaliano DF. On the estimation of hospital cost functions. J Health Econ. 1987;6: 305–318. doi: 10.1016/0167-6296(87)90018-x 10285440

[32] R Core Team. R: A language and environment for statistical computing. [Software] R Foundation for Statistical Computing, Vienna, Austria. [Software] 2020 [cited: 2020 Nov 10–2020 Feb 08] Available from: https://www.R-project.org/.

[33] Croissant Y, Millo G. Panel Data Econometrics in R: The plm Package. J Stat Softw. 2008; 27(2), 1–43. doi: 10.18637/jss.v027.i02 (URL: 10.18637/jss.v027.i02). [also a software available from R CRAN].

[34] Millo G. Robust Standard Error Estimators for Panel Models: A Unifying Approach. J Stat Softw. 2017; 82(3): 1–27. doi: 10.18637/jss.v082.i03

[35] Petrie A. regclass: Tools for an Introductory Class in Regression and Modeling. R package version 1.6. [Software] 2020 [cited: 2021 Jan 27] Available from: https://CRAN.R-project.org/package=regclass.

[36] Zeileis A, Hothorn T. Diagnostic Checking in Regression Relationships. R News. 2002; 2(3):7–10. URL https://CRAN.R-project.org/doc/Rnews/.

[37] Zeileis A. Econometric Computing with HC and HAC Covariance Matrix Estimators. J Stat Softw. 2004;11(10): 1–17. doi: 10.18637/jss.v011.i10 (URL: 10.18637/jss.v011.i10).

[38] Zeileis A. Object-Oriented Computation of Sandwich Estimators. J Stat Softw. 2006;16(9):1–16. doi: 10.18637/jss.v016.i09 (URL: 10.18637/jss.v016.i09).

[39] Zeileis A, Köll S, Graham N. Various Versatile Variances: An Object-Oriented Implementation of Clustered Covariances in R. J Stat Softw. 2020; 95(1): 1–36. doi: 10.18637/jss.v095.i01 (URL: 10.18637/jss.v095.i01).

[40] Dormann CF, Elith J, Bacher S, Buchmann C, Carl G, Carré G, et al. Collinearity: a review of methods to deal with it and a simulation study evaluating their performance. Ecography. 2013; 36: 27–46. doi: 10.1111/j.1600-0587.2012.07348.x

[41] O’Brien RM. A Caution Regarding Rules of Thumb for Variance Inflation Factors. Quality & Quantity. 2007; 41:673–690. doi: 10.1007/s11135-006-9018-6

[42] Stańdo-Górowska H. Funkcja kosztów operacyjnych szpitala i jej zastosowanie w procesie zarządzania., Nierówności Społeczne a Wzrost Gospodarczy. 2011; 20:383–392.

[43] Talarska M. Rachunek kosztów w szpitalu publicznym. Prace Naukowe Uniwersytetu Ekonomicznego we Wrocławiu. 2011; 182: 524–531.

[44] Kister A. Specyfika kosztów pośrednich w kontekście działalności szpitala publicznego. Zeszyty Naukowe Politechniki Częstochowskiej Zarządzanie. [Internet] 2018; 30: 89–97. [Cited: 2021 Jan 12] Available from: http://zim.pcz.pl/znwz/files/z30/8.pdf.

[45] Kludacz-Alessandri M. Wpływ stopnia komputeryzacji szpitala na jakość kalkulacji kosztów świadczeń zdrowotnych. Studia i Prace Kolegium Zarządzania i Finansów. 2017; 157: 107–128.

[46] Nojszewska E, Sielska A. Sytuacja finansowa szpitali powiatowych a niezapłacenie przez NFZ za wykonane świadczenia. In: Bartkowiak R, Matusewicz M, editors. Nowe wyzwania w naukach o gospodarce. Warszawa: Oficyna Wydawnicza SGH; 2020. p. 233–246. doi: 10.1016/j.msard.2020.102265

